# Enhancing diversity in medical education: Bridging gaps and building inclusive curricula

**DOI:** 10.1111/medu.15619

**Published:** 2025-02-06

**Authors:** Aimee Marie Charnell, Caitriona A. Dennis

**Affiliations:** ^1^ Leeds Institute of Medical Education University of Leeds Leeds UK; ^2^ Mid Yorkshire NHS Trust Wakefield UK; ^3^ Health Professions Education Unit Hull York Medical School York UK

## Abstract

This commentary highlights challenges embedding diversity in medical education, urging consistent curricula, diverse patient representation, and collaboration to better prepare students for serving varied patient populations.

In this issue of Medical Education, Malik et al. offer a report on general practitioner (GP) tutors' efforts to recruit diverse volunteer patients for medical student placements.^1^ Their observations, which highlight time and resource constraints and the prioritisation of clinical symptoms to fulfil curriculum requirements, provide insights into the challenges of developing curricula and educational practices embodying equity, diversity and inclusion. This commentary explores how clinician and patient diversity influences current undergraduate teaching environments. Diversity impacts student learning experiences, and as such, there needs to be a consistent approach facilitated through staff training and stakeholder collaboration.

The evolution of equity, diversity and inclusivity in medical education is dynamic and ongoing. Medical education has made significant progress in expanding access to women, racial minorities and individuals from lower socio‐economic backgrounds, fostering greater inclusivity and diversity. Frameworks, such as those from the Medical Schools Council, may support medical schools in creating increasingly inclusive environments, considering elements of diversity listed in the 2010 Equality Act.^2^, ^3^

The evolution of equity, diversity and inclusivity in medical education is dynamic and ongoing.

Medical schools' efforts and support in educating students from diverse backgrounds ensure that graduating doctors increasingly reflect the diversity of the patients they serve. This diversity positively impacts healthcare provision because it is well‐established that diverse doctors lead to benefits such as better quality care and patient satisfaction.^4^, ^5^

Medical schools' efforts and support in educating students from diverse backgrounds ensure that graduating doctors increasingly reflect the diversity of the patients they serve.

However, despite the increasing diversification of doctors, achieving adequate patient diversity in educational patient cases remains challenging. In any population, patients vary in sex, gender, sexuality, race, religion, weight, disability, occupation, education and wealth. Nevertheless, the diversity of clinical‐case teaching does not always reflect the variation of patients seen within clinical practice.^6^

Although understanding the specific needs of diverse patient groups is vital, there is no common conceptual understanding of diversity within medical curricula, and teaching is often variable in content and depth.^7^ This lack of common understanding hinders the development of inclusive learning environments for students in medical schools and higher education institutions. Although policies and strategies promote inclusive practices, students still experience inconsistencies.^8^, ^9^

As a case in point, consider the divergence between medical students often beginning to learn about anatomy, physiology and pharmacology by considering an ‘average’ 70‐kg man and an exercise a lecturer asked us to perform. In this lesson, he asked us to stand up. He then asked the females to sit, followed by males under 50 kg and over 90 kg. He called out increasingly closer weights until when, between 69 and 71 kg, he was left with just one male standing in a class of almost 250. Just one student closely represented the 70‐kg man. This basic example of diversity within a small student group mirrors the diversity found on a broader scale.

Interestingly, Malik et al.'s study highlights that one of the main challenges in focused patient recruitment for teaching is related to cultural diversity. As inclusive practices in medical education evolve, it becomes clear that various characteristics, perspectives and experiences make individuals unique. Therefore, educators should review teaching materials and resources to ensure they reflect the full spectrum of diversity, providing medical students with opportunities to interact with a truly diverse patient community. To achieve this, conducting an audit of curricula is fundamental and can be guided by existing frameworks and active inclusion initiatives.^2^, ^9^

Therefore, educators should review teaching materials and resources to ensure they reflect the full spectrum of diversity, providing medical students with opportunities to interact with a truly diverse patient community.

Guidelines from regulatory bodies such as the General Medical Council (GMC) in the United Kingdom and the Accreditation Council for Graduate Medical Education (ACGME) in the United States emphasise the need to understand and thus recruit patients with diverse characteristics to enhance student awareness of diversity and inclusion.^10^, ^11^ However, recruiting for diversity presents additional challenges since general case management often remains the primary focus in speciality placements due to clinical curricula priorities. Significant work is required to ensure faculty and students recognise and understand the varying presentations in diverse populations while balancing this with other curriculum demands.

Malik et al. recognised various layers of recruitment complexities, including medical schools' efforts to recruit diverse volunteer patients in primary care placements. Although public willingness exists to participate in university education, institutions need supportive infrastructures.^12^ Recruitment may involve enhancing relationships with patient communities, encouraging opportunistic patient recruitment, raising tutor awareness of inclusive educational practices and incorporating diversity‐focused teaching.^13^ Creating communities of practice and lived experience networks focusing on patient volunteers can support developing infrastructures to promote patient involvement.^14^ Since undergraduate medical education spans academic and clinical environments, collaborating with patients and other stakeholders will provide different perspectives of inclusivity within care and create authentic, multifaceted learning environments.^15^, ^16^

With a collaborative approach to education, undergraduate medical students can gain authentic experiences and encounter various degrees of diversity during each clinical rotation. During their primary care experience, students can be supported in recognising diversity, as their GP tutors will have insights into the composition and needs of their patient population. Additionally, community GP tutors who are more diverse and reflective of the student body may have lived experiences and insights that make them effective educational role models.^16^ However, patient populations and diversity still vary between GP practices, which medical schools should consider. This may mean rotating students through diverse practices and ensuring that teaching materials and policies focus on diversity. Faculty planning student clinical rotations should conduct diversity audits and work with emerging inclusion frameworks.

However, patient populations and diversity still vary between GP practices, which medical schools should consider. This may mean rotating students through diverse practices and ensuring that teaching materials and policies focus on diversity.

In conclusion, despite significant advancements, more work is needed to create an inclusive medical curriculum that reflects the full spectrum of diversity. The challenges highlighted by Malik et al. emphasise the need for ongoing efforts to develop genuinely inclusive curricula, including recruiting diverse volunteer patients and balancing clinical teaching priorities with diversity goals. By fostering collaboration among educators, clinicians and regulatory bodies, plus implementing diversity‐focused audits and inclusive teaching frameworks, medical schools can better prepare students to meet the needs of diverse patient populations. As we continue to advance our understanding and implementation of diversity in medical education, we must ensure that all students can learn from and interact with a wide range of patient experiences. This commitment to equity and representation will enhance student learning outcomes and improve the quality and inclusivity of healthcare delivery.

By fostering collaboration among educators, clinicians and regulatory bodies, plus implementing diversity‐focused audits and inclusive teaching frameworks, medical schools can better prepare students to meet the needs of diverse patient populations.

## AUTHOR CONTRIBUTIONS

**Aimee Marie Charnell:** Conceptualization; writing – original draft; writing – review and editing. **Caitriona A. Dennis:** Conceptualization; writing – original draft; writing – review and editing.

## CONFLICT OF INTEREST STATEMENT

None declared.

## ETHICS STATEMENT

Not required.
